# Cancer incidence in patients with type 2 diabetes mellitus: a population-based cohort study in Shanghai

**DOI:** 10.1186/s12885-015-1887-4

**Published:** 2015-11-05

**Authors:** Hui-Lin Xu, Hong Fang, Wang-Hong Xu, Guo-You Qin, Yu-Jie Yan, Bao-Dong Yao, Nai-Qing Zhao, Yi-Nan Liu, Fen Zhang, Wei-Xi Li, Na Wang, Jie Zhou, Jin-Ling Zhang, Li-Yun Zhao, Lun-Qiang Li, Yan-Ping Zhao

**Affiliations:** 1 School of Public Health, Fudan University, 138 Yi Xue Yuan Road, Shanghai, 200032 People’s Republic of China; 2 Shanghai Minhang Center for Disease Control and Prevention, 965 Zhong Yi Road, Shanghai, 201101 People’s Republic of China

**Keywords:** Type 2 diabetes mellitus, Cancer incidence, Cohort study

## Abstract

**Background:**

Type 2 diabetes mellitus (T2DM) has been suggested to increase the risk of cancers. The aim of this study was to investigate the risk of common cancers in Chinese patients with T2DM.

**Methods:**

A population-based retrospective cohort study including 36,379 T2DM patients was conducted in Minhang District of Shanghai, China, during 2004 to 2010. All T2DM patients were enrolled from the standardized management system based on local electronic information system. Newly-diagnosed cancer cases were identified by record-linkage with the Shanghai Cancer Registry. Standardized incidence ratios (SIR) and 95 % confidence interval (CI) were used to estimate the risk of cancers among T2DM patients.

**Results:**

Overall crude incidence rate (CIR) of cancers was 955.21 per 105 person-years in men and 829.57 per 105 person-years in women. Increased risk of cancer was found in both gender, with an SIR being 1.28 (95 % CI = 1.17–1.38) in men and 1.44 (95 % CI =1.32–1.55) in women. Increased risk of colon (SIR = 1.97; 95 % CI = 1.49 to 2.46), rectum (1.72; 1.23 to 2.21), prostate (2.87; 2.19 to 3.56), and bladder cancers (1.98, 1.28 to 2.68) were observed in men and elevated risk of colon (1.67; 1.25 to 2.08), breast (1.66; 1.38 to 1.95), and corpus uteri cancers (2.87; 2.03 to 3.71) were observed in women.

**Conclusions:**

Our results indicate that Chinese patients with T2DM may have an increased risk of some cancers, and the increase may vary by sub-sites of cancers.

## Background

Type 2 diabetes mellitus (T2DM) is one of the major public health problems around the world. Over past decades, the prevalence of T2DM has been increasing rapidly in Asian populations [1]. In China, the prevalence of diabetes and pre-diabetes reached 9.7 and 15.5 %, respectively, in adults at age of 20 years or above in 2008, which means that 92 million Chinese adults were with diabetes and 148 million with pre-diabetes in the country [2].

Epidemiologic evidence is accumulating on the association between T2DM and cancers, particularly for cancers of colorectum [3–5], liver [6, 7], breast [8, 9], bladder [10], kidney [11], and prostate [12]. However, the results are not consistent [13–17], and the associations differ by race and ethnicity [18]. To elucidate the associations of T2DM with the risk of cancers is particularly important in China, because, with the huge population and high prevalence of T2DM, even a small increase in the cancer risk might have a significant public health impact at the population level for the country.

The present study was specifically designed to reveal the possible associations of T2DM with overall and sub-sites of specific cancers in Chinese adults by a population-based risk analysis.

## Methods

### Data sources

Based on the Chinese National Diabetes Prevention Guide, standardized management for diabetes patients, as a basic community health service, has been carried out since 2004 in Minhang district, an administrative division with 1,000,000 residents of Shanghai, China. The patients enrolled in the management system included: 1) part of prevalent T2DM patients; 2) newly-diagnosed T2DM patients from local comprehensive hospitals, 3) newly-diagnosed T2DM patients from regular health check-up programs for all local residents over 60 years provided by community health service centers (CHSC), and 4) newly-diagnosed T2DM patients from free screening for high risk group with specific symptoms or claims provided by CHSC. Due to that the T2DM patients diagnosed in hospitals out of Minhang district were not routinely enrolled for standardized management, most T2DM patients registered in the system were from local hospitals, which accounted for about 90 % of all newly-diagnosed patients in local hospitals each year.

The standardized management referred to regular following-up of patients carried out by General Practitioners. According to the Chinese National Diabetes Prevention Guide, the frequency of following-up and health care service provided to the patients were determined and standardized according to the patient’s clinical conditions. The patients with fasting blood glucose < 7.0 mmol/L were followed up every 3 months by measuring blood pressure and testing levels of blood glucose, glycosylated hemoglobin and urine protein, and the patients with fasting glucose > 7.0 mmol/L were followed up once per month. During the 7-year period from 2004 to 2010, a total of 36,379 diabetes patients were enrolled and followed-up. All these data was recorded as electronic health records (eHR) and transmitted into the eHR database. Using the unique identification card number of each individual, the incident cancer cases were identified through record-linkage of eHR system with Cancer Registry System in Minhang district, which is part of the Shanghai Cancer Registry system [19, 20]. The ICD-10 codes were assigned to each cancer case by the type of cancers such as Stomach (C16), Colon (C18), Rectum (C19-C20), Liver (C22), Pancreas (C25),Trachea / bronchus and lung (C33-C34), Brain/central nervous system (C70-72), Prostate (C61), Kidney (C64), Bladder (C67), Breast (C50), Corpus uteri (C53) and Thyroid (C73).

Datum of all subjects was acquired based on local basic community health service. Verbal informed consent was obtained from all these enrolled patients. Ethics approval was granted from the Institutional Review Board of Minhang Center for Disease Control and Prevention (NO: EC-P-2012-002).

### Statistical analysis

Person-years (PY) of follow-up were calculated from the date that T2DM was first diagnosed to the date on which common cancer was diagnosed, or death date, or the December 31, 2010, whichever occurred first. Crude incidence rate (CIR) for cancers was calculated by the number of incident cancer cases divided by the number of observed person-years. The age-standardized rate (ASR [W]) was calculated using the World Standard Population.

The standardized incidence ratio (SIR) and its 95 % confident interval (95 % CI) were used to estimate the risk of common cancer in patients with T2DM. SIRs of cancers were determined by comparing the observed and expected number of cancer cases among T2DM patients, in which the latter was calculated by applying the age-specific incidence rates in the general population.

$$ SIR=\frac{the\kern0.5em  number\kern0.5em  of\kern0.5em  observed\kern0.5em  cancer\kern0.5em  cases}{the\kern0.5em  number\kern0.5em  of\kern0.5em  \exp ected\kern0.5em  cancer\kern0.5em  cases} $$

The 95 % CI of SIR was calculated using the following formula:

$$ SIR\pm 1.96{S}_e $$

Where

$$ S{}_e=\raisebox{1ex}{$ SIR$}\!\left/ \!\raisebox{-1ex}{$\sqrt{number\kern0.5em  of\kern0.5em  observed\kern0.5em  cases}$}\right. $$

*U* test based on Poisson distribution was applied to compare the CIRs of cancers between prevalent and newly-diagnosed T2DM patients by using the following formula:

$$ u=\frac{\raisebox{1ex}{${X}_1$}\!\left/ \!\raisebox{-1ex}{${n}_1$}\right.-\raisebox{1ex}{${X}_2$}\!\left/ \!\raisebox{-1ex}{${n}_2$}\right.}{\sqrt{X_1/{n_1}^2+{X}_2/{n_2}^2}} $$

X_1_ and *X*_2_ represent the number of cancer cases in prevalent and newly-diagnosed T2DM patients, and n_1_ and n_2_ represent person-years of observation for prevalent and newly-diagnosed T2DM patients, respectively.

All tests were two sided. *P* value less than 0.05 was considered as statistical significant.

## Results

Between Jan 1, 2004 and Oct 31, 2010, 36,379 T2DM patients were enrolled and followed-up regularly through eHR system in Minhang district of Shanghai, China. The characteristics of the T2DM patients are shown in Table 1. Of the 36,379 T2DM patients, 16,166 (44.4 %) were men and 20,213 (55.6 %) were women, with mean age being 58.44 and 59.37 years, respectively. After 136,187 person-years of following-up, 1205 cancer cases were identified through record-linkage of eHR system with the Cancer Registry System. The overall crude incidence rate (CIR) of cancers was 955.21 per 10^5^ person-years in men and 829.57 per 10^5^ person-years in women, with ASR (W) being 289.69 and 246.22 per 10^5^ person-years, respectively. The top ten cancer subtypes by CIR were Stomach, Colon, Rectum, Liver, Pancreas, Trachea, bronchus and lung, Brain, central nervous system, Prostate, Bladder, and Kidney cancer in men, and were Stomach, Colon, Rectum, Liver, Pancreas, Trachea/bronchus and lung, Brain, central nervous system, Breast, Corpus uteri, and Thyroid cancer in women, as shown in Table 2.

**Table 1:** Baseline description of the diabetes cohort

Variable	Men (*N* = 16166)		Women (*N* = 20213)	
Follow-up time (Median, years)	3.70	Range: 1–7	3.78	Range: 1–7
Age (mean, years)	58.44	Range: 21–94	59.37	Range: 21–95
Calendar year of enrolment (N, %)				
2004	755	4.67	978	4.84
2005	514	3.18	689	3.41
2006	2832	17.52	3940	19.49
2007	4469	27.64	5563	27.52
2008	5303	32.80	6532	32.32
2009	2009	12.43	2253	11.15
2010	284	1.76	258	1.28

**Table 2:** CIRs and ASRs of cancers in patients with T2DM

	Men (*N* = 16166)			Women (*N* = 20213)		
Subtypes	No. of cancer cases	CIR^a^	ASR^b^ (world)	No. of cancer cases	CIR	ASR (world)
Stomach	74	123.58	35.22	49	64.22	14.88
Colon	63	105.21	34.91	63	82.56	19.24
Rectum	48	80.16	21.49	30	39.32	10.09
Liver	51	85.17	23.43	24	31.45	10.25
Pancreas	24	40.08	11.94	18	23.59	6.20
Trachea, bronchus and lung	62	103.54	26.12	56	73.39	18.59
Brain, central nervous system	9	15.03	4.11	24	31.45	7.93
Prostate	68	113.56	35.93			
Kidney	16	26.72	7.36	9	11.79	5.43
Bladder	31	51.77	16.84			
Breast				132	172.99	53.40
Corpus uteri				45	58.97	24.74
Thyroid	3	5.01	1.77	26	34.07	19.56
Others	123	205.40	70.57	157	205.76	55.91
Total	572	955.21	289.69	633	829.57	246.22

^a^CIR: crude incidence rate (per 100000 person years)

^b^ASR: Age-standardized rate (per 100000 person years) calculated using World Standard Population data

SIRs of common cancers in T2DM patients by gender and subtypes of cancers are shown in Table 3. An increased risk of overall cancer in T2DM patients were found in both men and women, with SIRs of 1.28 (95 % CI: 1.17–1.38) and 1.44 (95 % CI: 1.32–1.55), respectively. For subtypes of cancer, a significant increased risk of colon (1.97; 1.49 to 2.46), rectum (1.72; 1.23 to 2.21), prostate (2.87; 2.19 to 3.56), and bladder cancers (1.98, 1.28 to 2.68) was observed in men and elevated risk of colon (1.67; 1.25 to 2.08), breast (1.66; 1.38 to 1.95) and corpus uteri cancers (2.87; 2.03 to 3.71) was observed in women. We did not find a significant association between T2DM and Stomach, Liver, Pancreas, Brain, Kidney cancers.

**Table 3:** SIRs of cancers in patients with type 2 diabetes by gender and subtype of disease

	Male (*N* = 16166)					Female (*N* = 20213)				
	No. of cancer cases		SIR	95 % CI		No. of cancer cases		SIR	95 % CI	
	Observed	Expected		Low	Upper	Observed	Expected		Low	Upper
Stomach	74	63.5	1.17	0.90	1.43	49	39.0	1.26	0.91	1.61
Colon	63	31.9	1.97	1.49	2.46	63	37.8	1.67	1.25	2.08
Rectum	48	27.9	1.72	1.23	2.21	30	23.5	1.28	0.82	1.73
Liver	51	42.5	1.20	0.87	1.53	24	22.0	1.09	0.65	1.53
Pancreas	24	18.7	1.28	0.77	1.80	18	18.8	0.96	0.51	1.40
lung	62	103.3	0.60	0.45	0.75	56	53.9	1.04	0.77	1.31
Brain	9	9.9	0.91	0.32	1.51	24	16.2	1.48	0.89	2.07
Prostate	68	23.7	2.87	2.19	3.56					
Kidney	16	10.9	1.47	0.75	2.19	9	6.3	1.42	0.49	2.35
Bladder	31	15.6	1.98	1.28	2.68					
Breast						132	79.4	1.66	1.38	1.95
Corpus uteri						45	15.7	2.87	2.03	3.71
Thyroid	3	4.2	0.71	0.09	1.51	26	20.2	1.29	0.79	1.79
Others	117	95.8	1.22	1.00	1.44	157	107.8	1.46	1.23	1.68
Total	572	447.9	1.28	1.17	1.38	633	440.7	1.44	1.32	1.55

The risk of cancers was not observed to increase in patients at all age groups. In men, an increased risk was found in patients at ages of 70- and 80- years, with SIRs of 1. 54 (95 % CI: 1.34–1.73) and 2.79 (95 % CI: 2.17–3.42), respectively. In women, an increased risk was also observed in older patients, with SIRs being 1.48 (95 % CI: 1.28–1.69) in patients at ages of 60- years, 1.79 (95 % CI: 1.56–2.03) in patients at ages of 70- years, and 2.20 (95 % CI: 1.61–2.78) in patients at ages of 80- years, respectively (as shown in Table 4).

**Table 4:** CIRs and SIRs of cancer in T2DM patients by age group

Age groups	Men					Women				
	No. of cancer cases		SIR	95 % CI		No. of cancer cases		SIR	95 % CI	
	Observed	Expected		Low	Upper	Observed	Expected		Low	Upper
30-	2	1.6	1.25	0.48	2.98	6	3.3	1.82	0.36	3.27
40-	16	23.0	0.70	0.35	1.04	34	39.0	0.87	0.58	1.16
50-	93	85.2	1.09	0.87	1.31	112	111.9	1.00	0.82	1.19
60-	142	152.8	0.93	0.78	1.08	204	137.4	1.48	1.28	1.69
70-	243	158.0	1.54	1.34	1.73	223	124.4	1.79	1.56	2.03
80-	76	27.2	2.79	2.17	3.42	54	24.6	2.20	1.61	2.78

In this study, we did not find a significant difference in age distribution between prevalent and newly-diagnoseddiabetes patients. However, the risk of cancers was significantly higher in prevalent diabetes patients (CIR = 494.37 per 10^5^ person-years) than in those newly-diagnosed (CIR = 991.04 per 10^5^ person-years). The difference was significant in both men (U = 5.06, *P* < 0.05) and women (U = 8.75, *P* < 0.05) (as shown in Table 5).

**Table 5:** Comparison on CIR of cancer between newly-diagnosed and prevalent T2DM patients

	Male				Female				Total			
	No. of T2DM	Person-years (PY) of follow-up	No. of cancer cases	CIR^#^	No. of T2DM	Person-years (PY) of follow-up	No. of cancer cases	CIR	No. of T2DM	Person-years (PY) of follow-up	No. of cancer cases	CIR
Newly-diagnosed D2TM patients	3899	12877	80	621.26	4875	16251	64	393.82	8774	29128	144	494.37
Prevalent D2TM patients	12267	47005	492	1046.70	15338	60054	569	947.48	27605	107059	1061	991.04
*U*-test	U = 5.06, *P* < 0.05				U = 8.75, *P* < 0.05				U = 6.44, *P* < 0.05			

^#^CIR: crude incidence rate (per 100000 person years)

## Discussion

The association between T2DM and the risk of common cancers has been extensively investigated in western countries. In China, however, only a few studies have been focused on the associations, and the findings were not consistent [15, 16]. In this study, based on local population-based registry data, we observed an increased risk of overall cancer in Chinese with T2DM. Our results are consistent with Wideroff et al’s report [13], in which diabetic patients in Denmark was found having 10 % increased risk of all types of cancers (SIR = 1.1). However, no significant association was found between T2DM and an incidence of cancer (RR = 0.99, 95 % CI = 0.90–1.09) in a UK study [17]. Ethnic discrepancy on biologic effect of T2DM may partly explain the inconsistency [18]. Moreover, due to the fact that the risks of cancers in diabetes patients may vary by sub-sites of cancers and the ranks of cancers differ across populations, it is necessary to compare the associations of T2DM with the risk of site-specific cancers in different populations.

One of the main findings in previous studies is that diabetes patients have an increased risk of colorectal cancer [17, 21, 22]. However, the increases in risk of colon and rectum cancers were found much different. Ren, et al. [23] reported a significant increased risk of colon cancer in male T2DM patients and a borderline significant risk of colon cancer in females, and did not find a significant association between T2DM and risk of rectal cancer in both sexes. The results in this study are sometime inconsistent with Ren, et al’s report. Our patients, both male and female, were more likely to have colon cancer. The increase in the risk of rectum cancer was also observed in both sexes, but the association reached significant only in male patients.

Our finding of an elevated risk of prostate cancer in male T2DM patients is consistent with the results reported by Tseng, et al. [24] and Will, et al. [25], but is not in line with some other studies. Evidence has been available for the null [26] and inverse associations between T2DM and prostate cancer [27, 28]. The result from a meta-analysis indicates that diabetes was associated with an increased risk of prostate cancer in Asians but the RR was greatly attenuated by adjusting for some confounders (unadjusted RR = 2.82, adjusted RR = 1.31) [29]. The mechanism underlying the association is unclear. It is possible that the difference in exposures to common risk factors such as tobacco smoking, obesity, and some psychology factors [30] and difference in genetic background [31–33] may contribute to the inconsistency. Moreover, average level of PSA was significantly lower in diabetes patients than in non-diabetes, as were as frequency of PSA screening [34], which may partly explain the inverse association between T2DM and prostate cancer.

For other types of cancers, we observed higher risks of breast and Corpus uteri cancers in females and bladder cancer in males, which is consistent with previous studies [15, 16]. A significant inverse association between diabetes and lung cancer was observed in men, which is also consistent with previous studies [16]. However, we did not find a significant association of T2DM with the risk of pancreatic and kidney cancers, which is not consistent with the positive association reported in several studies [35, 36]. Inadequate statistical power due to small sample size may explain the results on rare cancers. Moreover, we did not take some potential confounders into consideration such as sedentary lifestyle, tobacco smoking and obesity, the shared risk factors of T2DM and certain cancers [37]. More studies are needed to distinguish the effect of diabetes from those of shared risk factors and diabetes management.

Many biological mechanisms may be involved in the elevated risks of overall or certain sub-sites cancers among T2DM patients. Usually, diabetic patients have hyperinsulinemia and are associated with reduced insulin sensitivity and compensatory hyperinsulinemia as well as an increased insulin-like growth factor (IGF)-1 level, which may stimulate cell proliferation in pancreas, colon, breast, and other organs. Insulin itself exerts a mitogenic effect on various tissues including breast cancer cell lines, which are usually oestrogen receptor positive [38]. In breast cancer, insulin induces aromatase activity and reduces sex hormone binding globulin (SHBG), leading to increased free oestrogen levels, which in turn increases mitogenicity [39]. Interestingly, breast cancer cells appear to have high levels of insulin receptors, compared to normal breast tissue [40]. Insulin may exert a mitogenic effect through insulin-like growth factor-1 (IGF-1) receptors. Prospective studies have shown that people with circulating IGF-1 have an increased risk of common epithelial cancers such as breast, colon and prostate cancer [41]. A recent large population based survey showed that women with high blood concentrations of IGF-1 are more likely to develop breast cancer, women with the highest concentration of IGF-1 were found to have a 28 % higher risk of developing breast cancer than women with the lowest concentration [OR 1.28 (95 % CI 1.14–1.44)] [42]. The direct link between diabetes and cancer through hyperinsulinemia, hyperglycemia and inflammation may explain the high risk of cancers in prevalent T2DM patients than in those newly-diagnosed. It has been suggested that duration of diabetes and use of exogenous insulin were associated with the cancer risk [30]. It seems that long-term exposure to insulin has direct relevance to the cancer risk.

### Strengths and limitations

The strengths of this study included its prospective study design, representative and relative large sample size of diabetes patients, confirmation system of diagnosis and high quality cancer registry system, which provide us a good opportunity to evaluate the possible effect of T2DM on cancer risk in Chinese population. Several limitations of the study should be mentioned. First, the small number of some site-specific cancers may lead to a low statistical power to detect a significant association. Therefore, we only focused on the top ten subtypes of cancers to ensure statistical power. Second, the average following-up time was rather short for many patients, which also may influence the statistical power. Moreover, due to the number of patients screened varied year by year, it is possible that the patients recruited in certain years were detected earlier while those in other years were not, which may lead to heterogeneity of the patient population. Third, although age and gender were controlled in calculation of SIR, we did not obtain and adjust other risk factors such as smoking, alcohol consumption, body mass index (BMI), physical activity and use of antidiabetic medicines, in which use of antidiabetic drug metformin has been associated with reduced risk of cancers [36] and BMI was reported to be associated with increased risk of both of T2DM and cancers [37]. Finally, diabetes is an under-diagnosed disease and some T2DM patients remained undiagnosed, which may lead to misclassification bias.

## Conclusion

In summary, the present study suggested that type 2 diabetes may increase the risks of overall cancers, particularly in older patients and prevalent patients. Diabetes is associated with an increased risk some cancers (colon, rectum, prostate, and bladder cancers in men and colon, breast, and corpus uteri cancers in women), and related with a reduced risk of lung cancer in men. For some other cancer sites, there appears to be no association possibly due to inadequate statistics power.
